# A Multicentric Randomized Trial to Evaluate the ROle of Uterine MANipulator on Laparoscopic/Robotic HYsterectomy for the Treatment of Early-Stage Endometrial Cancer: The ROMANHY Trial

**DOI:** 10.3389/fonc.2021.720894

**Published:** 2021-09-10

**Authors:** Salvatore Gueli Alletti, Emanuele Perrone, Camilla Fedele, Stefano Cianci, Tina Pasciuto, Vito Chiantera, Stefano Uccella, Alfredo Ercoli, Giuseppe Vizzielli, Anna Fagotti, Valerio Gallotta, Francesco Cosentino, Barbara Costantini, Stefano Restaino, Giorgia Monterossi, Andrea Rosati, Luigi Carlo Turco, Vito Andrea Capozzi, Francesco Fanfani, Giovanni Scambia

**Affiliations:** ^1^Division of Gynecologic Oncology, Fondazione Policlinico Universitario A. Gemelli IRCCS, Rome, Italy; ^2^Institute of Obstetrics and Gynecology, Universita’ Cattolica del Sacro Cuore, Rome, Italy; ^3^Department of Human Pathology of Adult and Childhood “G. Barresi,” Unit of Gynecology and Obstetrics, University of Messina, Messina, Italy; ^4^STAR Center (Statistics Technology Archiving Research), Fondazione Policlinico Universitario Agostino Gemelli-IRCCS, Rome, Italy; ^5^Department of Gynecologic Oncology, ARNAS Civico Di Cristina Benfratelli, Università di Palermo, Palermo, Italy; ^6^Department of Obstetrics and Gynecology, AOUI Verona, Università di Verona, Verona, Italy; ^7^Division of Obstetrics and Gynecology, Università degli studi di Messina, Policlinico G. Martino, Messina, Italy; ^8^Division of Gynecologic Oncology, Gemelli-Molise, Università Cattolica del Sacro Cuore, Campobasso, Italy; ^9^Department of Maternal and Child Health, University-Hospital of Udine, Udine, Italy; ^10^Department of Gynecology and Obstetrics of Parma, University of Parma, Parma, Italy

**Keywords:** hysterectomy, endometrial cancer, robotic hysterectomy, laparoscopic hysterectomy, minimally invasive hysterectomy, uterine manipulator

## Abstract

**Objective:**

This prospective randomized trial aimed to assess the impact of the uterine manipulator in terms of lymph vascular space invasion (LVSI) in patients undergoing minimally invasive staging for early-stage endometrial cancer.

**Methods:**

In this multicentric randomized trial, enrolled patients were randomly allocated in two groups according to the no use (arm A) or the use (arm B) of the uterine manipulator. Inclusion criteria were G1-G2 early-stage endometrial cancer at preoperative evaluation. The variables collected included baseline demographic characteristics, perioperative data, final pathology report, adjuvant treatment, and follow-up.

**Results:**

In the study, 154 patients (76 in arm A and 78 in arm B) were finally included. No significant differences were recorded regarding the baseline characteristics. A statistically significant difference was found in operative time for the laparoscopic staging (p=0.005), while no differences were reported for the robotic procedures (p=0.419). The estimated blood loss was significantly lower in arm A (p=0.030). No statistically significant differences were recorded between the two study groups in terms of peritoneal cytology, LVSI (p=0.501), and pattern of LVSI (p=0.790). No differences were detected in terms of overall survival and disease-free survival (p=0.996 and p=0.480, respectively). Similarly, no differences were recorded in the number of recurrences, 6 (7.9%) in arm A and 4 (5.2%) in arm B (p=0.486). The use of the uterine manipulator had no impact on DFS both at univariable and multivariable analyses.

**Conclusions:**

The intrauterine manipulator does not affect the LVSI in early-stage endometrial cancer patients undergoing laparoscopic/robotic staging.

**Clinical Trial Registration:**

https://clinicaltrials.gov, identifier (NCT: 02762214)

## Introduction

Nowadays, it is common knowledge that minimally invasive surgery represents the standard approach for the staging of early-stage endometrial cancer (EC). Based on the risk factors, other staging procedures, as well as sentinel node mapping and/or pelvic and para-aortic lymphadenectomy, can be safely performed (1–3). If laparoscopy is classically associated with lower intraoperative blood loss and postoperative complications rate (4–6), robotic surgery has established itself as a preferable surgical approach for obese EC patients (7, 8).

In this context, the intrauterine manipulator has had an important role in minimally invasive hysterectomy since its diffusion, more than two decades ago. The possibility to expose the surgical field, giving the right tension to the superficial and the retroperitoneal anatomy, was immediately considered advantageous assistance (9). However, only once the laparoscopic approach was systematically adopted in EC staging, its role has been debated in terms of influence on oncological variables like peritoneal cytology and lymph vascular space invasion (LVSI).

Elthabbakh et al., in a prospective study, reported that laparoscopic surgery does not increase the positive peritoneal cytology in women with endometrial carcinoma (10), but its clinical significance remained controversial (11–13). Besides, the lymph vascular space invasion (LVSI) has been recognized to be an independent risk factor for pelvic lymph node recurrence, and it is found in about 15% of low-risk endometrial cancer patients (14–16).

Concerningly, the use of the uterine manipulator during laparoscopic EC staging has also been investigated as a factor affecting both the rate of positive peritoneal cytology and the risk of LVSI.

The possible role in polluting the peritoneal cytology has been linked to the retrograde seeding of tumor cells into the peritoneal cavity, consequent to the increased intrauterine pressure (17, 18). Furthermore, the higher rate of lymph vascular invasion, well described in EC patients, has been demonstrated to be the result of the grossing process and mechanical transport of manipulator-disrupted tumor into the vascular spaces (19–21).

Contrary to these preliminary reports, recent literature reported that the use of the uterine manipulator does not significantly affect the LVSI and the cytology and has no negative impact on the oncological outcomes (22, 23). However, definitive conclusions on the possible advantages and oncological safety of the use of the uterine manipulator in EC are still awaited (24).

This prospective randomized trial aimed to assess the impact of the uterine manipulator in terms of LVSI in patients undergoing minimally invasive (laparoscopic/robotic) staging for clinically presumed early-stage G1-G2 endometrial cancer.

## Methods

This is a multicentric Italian prospective 1:1 randomized clinical trial designed and coordinated by the Division of Gynecologic Oncology of Fondazione Policlinico Universitario A. Gemelli IRCCS of Rome. Four other Italian tertiary care centers took part in the study. The trial was approved by the local ethics committees (protocol number: 05152016) and was registered at https://clinicaltrials.gov (NCT:02762214). Between October 2015 and December 2017, all patients undergoing minimally invasive surgery, either laparoscopic or robotic, for preoperative early-stage EC were considered eligible.

The inclusion criteria were the following: G1-G2 early-stage EC at preoperative workup, age >18 and <80 years, and clinical condition fitting for minimally invasive treatment. An American Society of Anesthesiology (ASA) Score >III, previous cancer conditions, intact hymen, previous pelvic radiotherapy were considered exclusion criteria.

Enrolled patients were randomly allocated in two arms according to the use (arm B—experimental arm) or no use (arm A—control arm) of the uterine manipulator. The randomization list was developed by a statistician according to a random sorting using a maximum allowable 9% deviation; no stratification was anticipated. The IUM used in all procedure was the Clermont-Ferrand model by Storz.

All patients signed the specific informed consent to be enrolled in the study. All the procedures were performed by seven surgeons with an experience of at least 100 minimally invasive hysterectomies for EC.

Study data were collected using REDCap electronic data capture tools hosted at https://redcap-irccs.policlinicogemelli.it and were managed by the Statistics Technology Archiving Research (STAR) Center of the Fondazione Policlinico Universitario A. Gemelli IRCCS. Variables collected included baseline demographic characteristics, perioperative data, final pathology report, adjuvant treatment, and follow-up.

Intraoperative complications were defined as any injury to the bowel, urinary tract, nerves, and vessels, an estimated blood loss (EBL) ≥500 ml, and any needs to reinstall/remove the manipulator. The postoperative complications were categorized in accordance with the Clavien-Dindo Classification (25). All patients received a follow-up examination according to the ESMO-ESGO-ESTRO guidelines (26). The primary objective of the study was to assess the effect of the uterine manipulator in terms of LVSI rate in the final pathological report after the MIS staging. The secondary endpoints were the evaluation of peritoneal cytology, perioperative and oncological outcomes [operative time (OT) calculated skin to skin, EBL, complication rate, conversion rate, disease-free survival (DFS), and overall survival (OS)] in relation with the usage or not of the intrauterine manipulator (IUM). Patients were followed up every 4 months for the first 2 years and every 6 months thereafter.

### Surgical Technique

#### Arm A: Hysterectomy Without Uterine Manipulator

The cervix is closed with a cross stitch before starting abdominal procedures, and two gauzes rolled up inside a glove are inserted into the vagina to obtain a cranial push of the uterus. The procedure consists in total extrafascial hysterectomy (27) based on the retroperitoneal preparation of the surgical spaces and identification of the main anatomical structures such as uterine arteries and ureters. Subsequently, the vesico-uterine septum is developed, and the vascular structures are coagulated and divided. At the time of the vaginal cutting, the same gauzes allow the colpotomy, the manipulation of the vaginal apex, and the maintenance of the pneumoperitoneum.

#### Arm B: Hysterectomy With Uterine Manipulator

The surgical procedure for Arm B consists of the same steps described for Arm A with the usage of the intrauterine manipulator installed before starting the surgical dissection. The anterior colpotomy is performed using the monopolar hook under the guidance of the vaginal valve of the Clermont Ferrand uterine manipulator.

Once the hysterectomy is carried out, the specimen is removed throughout the vagina. The vaginal vault is then closed laparoscopically or vaginally with a 0 absorbable multifilament running suture.

In the study population, the lymph nodal assessment was performed, when indicated, following the NCCN guidelines (28). Pelvic cytology has been collected before starting the surgical procedure (“peritoneal washing 1”) and at the end of colporrhaphy using 200 ml of saline solution (“peritoneal washing 2”).

### Sample Size and Statistical Analysis

The study was designed to evaluate if the use of intrauterine manipulator was associated with a higher impact of LVSI rate. According to literature data (29), assuming a 10 and 25% of LVSI in the control and experimental arm, respectively, with an alpha error = 0.05, a statistical power of 80%, and a 1:1 ratio, the estimated sample sizes for a two-sample one-sided proportions test was 152 patients (76 per arm).

Patient’s characteristics were described as absolute frequency and percentage for nominal variables and as median (min-max) for continuous variables. Comparisons between the two arms were made with Mann-Whitney test or t Student’s test for continuous variables and χ2 or Fisher exact test for nominal variables, as appropriate. The normality of continuous variables was assessed with Shapiro-Francia test.

Survival analysis was performed both in terms of disease-free survival (DFS) and overall survival (OS). DFS was defined as the time elapsed from first diagnosis to recurrence/progression or last follow-up, while OS was defined as the time from first diagnosis to death for disease or last follow-up. Median follow-up was calculated according to the inverted Kaplan-Meier technique. OS and DFS curves were estimated by Kaplan-Meier product limit method and compared by log-rank test (30). For DFS, Cox proportional hazards models were used to assess treatment effect at univariable and multivariable analysis. Stage (I *vs* II-III-IV), histology (endometrioid *vs* serous), myometrial invasion (no infiltration or <50 *vs >*50%), postoperative tumor size (diameter ≤3.5 *vs >*3.5 *cm*), LVSI (negative *vs* positive) were covariates included in the analysis due to their clinical relevance. All estimates were presented with two-sided 95% Confidence Intervals (CIs).

Statistical analysis had been performed using STATA software (STATA/IC 13.0 for Windows, College Station, TX, USA, StataCorp LP). Two-sided tests were used, and the significance level was set at p < 0.05. No imputation was carried out for missing data.

## Results

**Figure 1:**
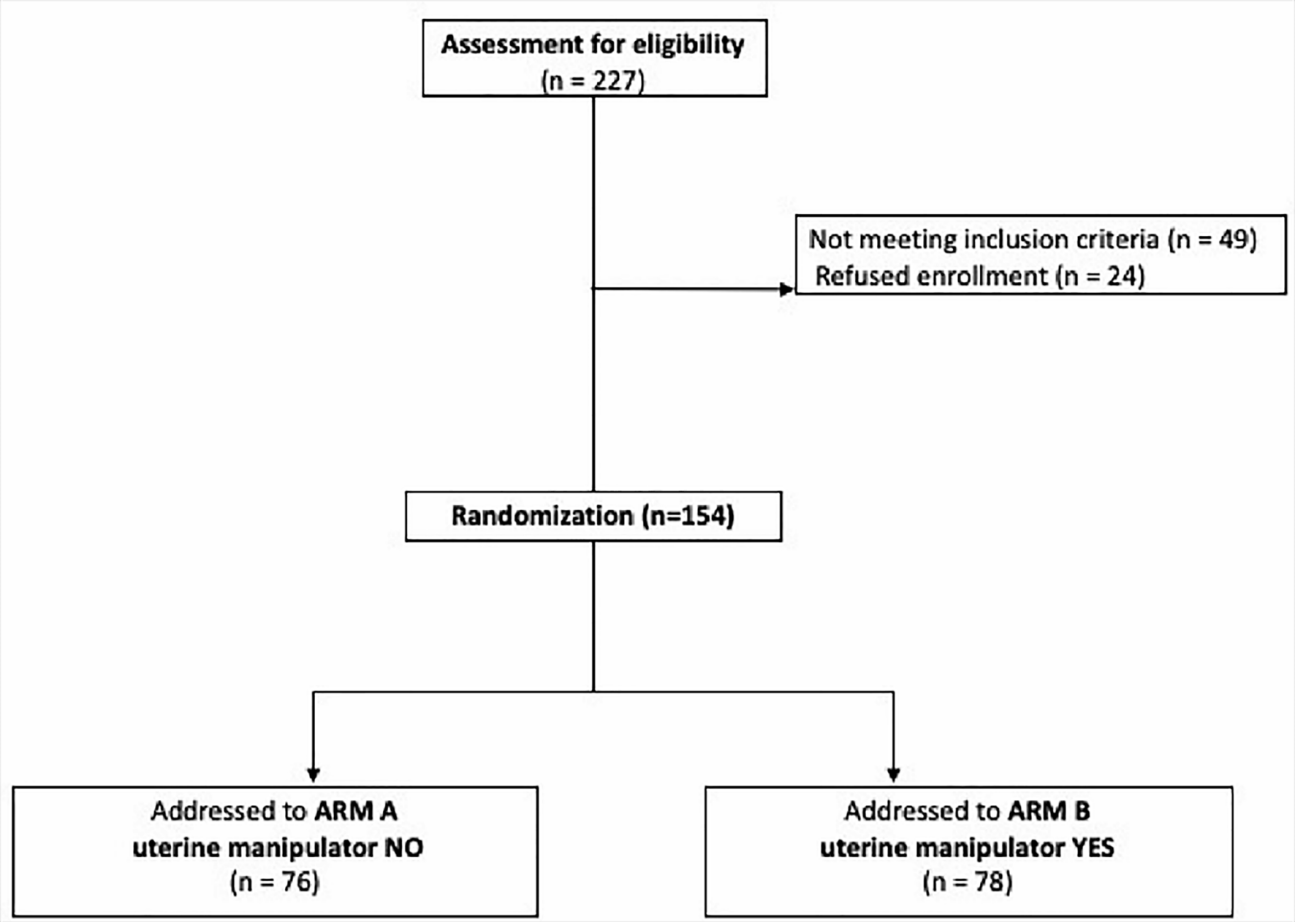
Consort diagram of the study.

During the study period, 227 patients were considered eligible for the study. Among them 24 refused to participate, and 49 did not meet the inclusion criteria. The remaining 154 patients met the inclusion criteria and were enrolled in the present study. Among them, 78 patients received surgery with the use of uterine manipulator (Arm B), and in 76 patients the IUM was not used (Arm A) (**Figure 1**).

Baseline demographic characteristics are summarized in **Table 1**. No significant differences were recorded between the two study arms in terms of body mass index (BMI) (overall median 28 kg/m2, range 19–46), previous abdominal surgery, and ASA score. Both study groups were similar also for previous cesarean section and parity. The only slightly statistically significant difference was reported for the age (p=0.045).

**Table 1:** Patient/Disease baseline characteristics.

Variables	All cases (n = 154)	Arm A: no IUM (n = 76)	Arm B: IUM (n = 78)	p value
Age, years	61 (31–81)	63 (36–81)	59.5 (31–77)	**0.045**
BMI, kg/m^2§^	28 (19–46)	28 (19–40)	27 (20–46)	0.490
Previous abdominal surgery^§^	71/151 (47)	40/76 (52.6)	31/75 (41.3)	0.164
Previous cesarean section^§^	28/151 (18.5)	15/76 (19.7)	13/75 (17.3)	0.704
ASA score^*^				0.145
I	23/131 (17.6)	9/68 (13.2)	14/63 (22.2)	
II	101/131 (77.1)	57/68 (83.8)	44/63 (69.8)	
III	7/131 (5.3)	2/68 (2.9)	5/63 (7.9)	
Parity^ŧ^	91/145 (62.8)	46/73 (63.0)	45/72 (62.5)	0.949
Preoperative and pathological findings				
Histotype				
Endometrioid	154 (100)	76 (100)	78 (100)	–
Grading				
1	65/130 (50.0)	35/65 (53.8)	30/65 (46.2)	0.380
2	65/130 (50.0)	30/65 (46.2)	35/65 (53.8)	
Maximum diameter at ultrasound^†^	25.5 (0–78)	28 (0–78)	24 (0–55)	0.151
Maximum diameter at MRI^‡^	20 (0–73)	23 (0–73)	13 (0–55)	0.059

Results are presented as n (%) or median (min-max) as appropriate. Bold font highlights statistically significant differences. IUM, intrauterine manipulator; BMI, body mass index; MRI, magnetic resonance imaging. ^§^Information available for 151/154 patients. *Information available for 131/154 patients. ^ŧ^Information available for 145/154 patients. Information available for 130/154 patients. ^†^Information available for 138/154 patients. ^‡^Information available for 57/154 patients.

Pathological baseline characteristics have been also recorded and are summarized in **Table 1**. In accordance with the inclusion criteria, all patients had a preoperative diagnosis of endometrioid EC, and no differences in the rate of grading between both arms were recorded. Both study groups were also similar in terms of maximum tumor diameter measured at pelvic ultrasound (overall median 25.5 mm, range 0–78 mm; p=0.151) and at magnetic resonance imaging (MRI) (overall median 20 mm, range 0–73 mm; p=0.059).

The perioperative data are shown in **Table 2**. Ninety-three procedures (60.4%) were performed by laparoscopy and 60 (39.0%) by robotic approach, and no differences in type of surgical approach were recorded between the two arms (p=0.566). We registered a shorter OT in arm A rather than in arm B [median OT arm A 140 min (range 50–300) *versus* median OT arm B 180 min (range 60–370); p=0.040). More in depth, stratifying OT according to the type of approach used, the statistically significant difference was found only for laparoscopy procedures [median OT 130 min (range 80–290) *versus* 170 min (range 70–370) in arm A and B respectively; p=0.003], while no differences were reported for OT of robotic procedures (p=0.419). The EBL was significantly lower in the control arm [median EBL 50 ml (range 0–250) and 50 ml (range 0–550) in arm A and B, respectively; p=0.030]. We reported one conversion to laparotomy (1.3%) in arm B for a vascular injury during pelvic lymph adenectomy and none in arm A (p=0.322).

**Table 2:** Perioperative variables.

Variables	All cases (n = 154)	Arm A: no IUM (n = 76)	Arm B: IUM (n = 78)	p value
Lymph nodal assessment				
Pelvic Lymph nodes^¶^	106/153 (69.3)	50/76 (65.8)	56/77 (72.7)	0.352
Sentinel Lymph nodes	29/106 (27.3)	12/56 (21.4)	17/59 (28.8)	0.481
Type of surgical approach				0.566
Laparoscopy	93 (60.4)	45 (59.2)	48 (61.5)	
Robotic	60 (39.0)	31 (40.8)	29 (37.2)	
Operation time (OT), min	150 (50–370)	140 (50–300)	180 (60–370)	**0.040**
OT for laparoscopic surgery, min	150 (70–370)	130 (80–290)	170 (70–370)	**0.003**
OT for robotic surgery, min	170 (50–300)	150 (50–300)	180 (60–270)	0.419
Estimated blood loss (EBL), ml^*^	50 (0–550)	50 (0–250)	50 (0–550)	**0.030**
EBL for laparoscopic surgery, min	50 (0–550)	50 (0–200)	50 (0–550)	**0.0002**
EBL for robotic surgery, min	50 (0–250)	50 (0–250)	50 (0–100)	0.101
Discharge time, days^§^	2 (1–3)	2 (1–3)	2 (1–3)	0.360
Conversion to laparotomy	1 (0.6)	0 (0)	1 (1.3)	0.322
Intraoperative complications	5 (3.2)	1 (1.3)	4 (5.1)	0.182
Early postoperative complications	7/151 (4.6)	5/74 (6.8)	2/77 (2.6)	0.270

Results are presented as n (%) or median (min-max) as appropriate. Bold font highlights statistically significant differences. IUM, intrauterine manipulator. ^¶^Information available for 153/154 patients. *Information available for 124/154 patients, 66 patients undergoing laparoscopic surgery and 46 patients robotic surgery. ^§^Information available for 151/154 patients.

The intraoperative complications were 5 (6.4%) in arm A and 1 (1.3%) in arm B. In particular, in the control arm, one lower third vaginal laceration was reported during the extraction of an enlarged uterus throughout the vagina. In the experimental arm we recoded two median/upper third vaginal lacerations; one vascular complication that, as above described, required a conversion to longitudinal laparotomy; one case of intraoperative removal of the IUM due to the inability to properly manipulate a strongly adherent uterus; one case of bladder lesion for accidental perforation of the upper third of the cervical canal with the IUM.

Moreover, five (6.7%) postoperative complications were registered in arm A: three vaginal cuff dehiscences (two Grade 3 and one Grade 1), one lymphocele (Grade 3), and one sepsis (Grade 2). Two postoperative lower urinary tract infection (Grade 2) were recorded in arm B (2.5%). No statistically differences were detected between the two groups in terms of intra- and postoperative complications (p=0.182 and p=0.270, respectively). The rate of lymph nodal assessment was similar between the two arms (p= 0.352). In particular, 29 patients received sentinel lymph-node mapping, and no differences were detected between the two groups (12 patients, 21.4% in group A; and 17, 28.8% in group B, p= 0.481).

As shown in **Table 3**, the final pathology reports were similar between the two groups. No statistically significant differences were detected in terms of lymph nodal status, histotype, grading, and stage. However, a G3 EC was diagnosed in 16/147 (10.9%) patients, a serous histotype in three (1.9%) cases (all G3) and a stage III-IV in eight (5.2%) cases (2/8 G3). The overall rate of upstaging was 5.2% (6.6 *versus* 3.8% in arm A and B, respectively; p=0. 445).

**Table 3:** Pathological findings.

Variables	All cases (n = 154)	Arm A: no IUM (n = 76)	Arm B: IUM (n = 78)	p value
Lymph nodal status				
Pelvic Lymph nodes^¶^				0.968
Negative	148/152 (97.4)	73/75 (97.3)	75/77 (97.4)	
Positive	4/152 (2.6)	2/75 (2.7)	2/77 (2.6)	
Histotype				0.545
Endometrioid	151 (98.1)	74 (97.4)	77 (98.7)	
Serous	3 (1.9)	2 (2.6)	1 (1.3)	
Grading^§^				0.875
G1	41/147 (27.9)	20/74 (27.0)	21/73 (28.8)	
G2	90/147 (61.2)	45/74 (60.8)	45/73 (61.6)	
G3	16/147 (10.9)	9/74 (12.2)	7/73 (9.6)	
Myometrial infiltration				0.341
No infiltration	4 (2.6)	2 (2.6)	2 (2.6)	
<50%	117 (76)	54 (71.1)	63 (80.8)	
>50%	33 (21.4)	20 (26.3)	13 (16.7)	
Maximum diameter, mm^*^	30 (0–90)	30 (0–90)	25 (0–85)	0.171
Lymph Vascular Space Invasion (LVSI)				0.501
Negative LVSI	121 (78.6)	58 (76.3)	63 (80.8)	
Positive LVSI	33 (21.4)	18 (23.7)	15 (19.2)	
Focal	28/33 (84.8)	15/18 (83.3)	13/15 (86.7)	0.790
Diffused	5/33 (15.2)	3/18 (16.7)	2/15 (13.3)	
Stage				0.867
IA	119 (77.3)	57 (75.0)	62 (79.5)	
IB	23 (14.9)	12 (15.8)	11 (14.1)	
II	4 (2.6)	2 (2.6)	2 (2.6)	
III–IV^◊^	8 (5.2)	5 (6.6)	3 (3.8)	
Cytological findings				
Pre-hysterectomy				–
Negative	154 (100)	76 (100)	78 (100)	
Positive	0 (0)	0 (0)	0 (0)	
Post-hysterectomy				–
Negative	154 (100)	76 (100)	78 (100)	
Positive	0 (0)	0 (0)	0 (0)	

Results are presented as n (%) or median (min-max) as appropriate. IUM, intrauterine manipulator. ^¶^Information available for 152/154 patients. ^§^Information available for 147/154 patients. *Information available for 150/154 patients. ◊ Two IIIA, one IIIB, four IIIC, and one IVB.

No positive cytological collections, either pre-hysterectomy or post-hysterectomy, were detected in the study population.

The analysis of the LVSI revealed no difference between patients undergoing hysterectomy with and without the IUM (p=0.501). The overall rate of positive LVSI was 21.4%. Moreover, the sub-analysis among the positive LVSI cases demonstrated that the IUM did not affect the pattern of lymphovascular spread (overall focal LVSI 84.8%, overall diffused LVSI 15.2%, p=0.790).

**Figure 2:**
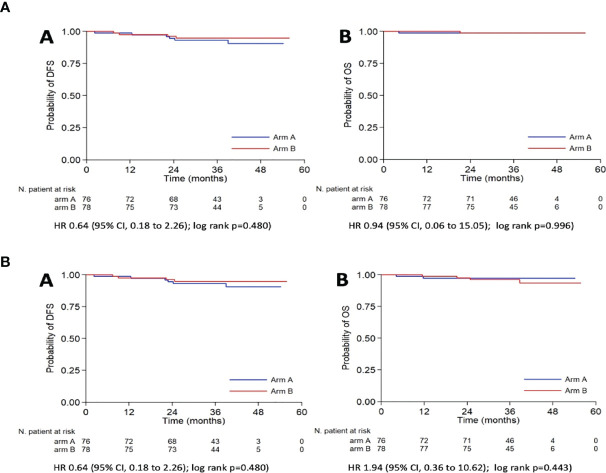
Kaplan-Meier curves for DFS **(A)** and OS **(B)** according to the use of IUM. (a) DFS, Disease Free Survival; OS, Overall survival; (b) DFS, Disease Free Survival; OS, Overall survival (death for any cause); IUM, Intrauterine manipulator; HR, Hazard Ratio; CI, Confidence Interval.

The oncological outcomes are summarized in **Table 4**. With a median follow-up of 38.7 months (CI: 37.1–40.8 months), no differences were detected in term of DFS and OS, as shown in **Figure 2A**.

**Table 4:** Oncological outcomes.

Variables	All cases (n = 154)	Arm A: no IUM (n = 76)	Arm B: IUM (n = 78)	p value
Median follow up, months (95% CI)^‡^	38.7 (37.1–40.8)	40 (36.5–42.2)	38.2 (35.0–40.6)	0.906^*^
Median OS, months	not reached	not reached	not reached	0.435^*^
Median DFS, months	not reached	not reached	not reached	0.480^*^
Deaths for any cause	6 (3.9)	2 (2.6)	4 (5.1)	0.423
Deaths for disease	2 (1.3)	1 (1.3)	1 (1.3)	0.985
Relapses	10 (6.5)	6 (7.9)	4 (5.1)	0.486
Site of relapse				0.383
Pelvic	3 (30.0)	3 (5.00)	0 (0)	
Lymph nodal	2 (20.0)	1 (16.7)	1 (25.0)	
Distant localization	2 (20.0)	1 (16.7)	1 (25.0)	
Multiple sites	3 (30.0)	1 (16.7)	2 (50.0)	
Adjuvant therapy				0.710
None	104/153 (68.0)	51/76 (67.1)	53/77 (68.8)	
RT	32/153 (20.9)	15/76 (19.7)	17/77 (22.1)	
CHT	17/153 (11.1)	10/76 (13.2)	7/77 (9.1)	

Results are presented as n (%) or median (min-max) as appropriate. CI, Confidence Interval; OS, Overall Survival; DFS, Disease-Free Survival; RT, Radiotherapy; CHT, Chemotherapy. ^‡^Calculated with inverse Kaplan-Meier method. *Log rank test. Three patients had pelvic relapse plus lymph nodal, carcinomatosis, and distant relapse respectively.

Similarly, no significant differences were reported in the rate of deaths for any cause, deaths for disease, and recurrences (p=0.423, p=0.985, and p=0.486, respectively) (**Figure 2B**).

In total, 10 (6.5%) recurrences have been registered during the follow-up period, six (7.9%) in arm A and four (5.1%) in arm B. In arm A, 50% of recurrences were vaginal relapses (3), one was lymph nodal, one extra-abdominal, and one multiple-site (carcinomatosis with sigmoidal involvement): except one patient with low-risk carcinoma, in the remaining five cases, a high-risk EC was diagnosed at final pathological examination. In arm B we recorded the following recurrences: one lymph nodal, one extra-abdominal, two multiple-site (vaginal + extra-abdominal and carcinomatosis); these patients had received a final pathological diagnosis of intermediate-high (2) and high-risk (2) tumors.

The postoperative management for all patients in both arms was substantially homogeneous; the 68% of the study population did not receive any adjuvant treatment.

At univariable analysis, tumor stage, postoperative tumor size (maximum diameter), and LVSI were independent predictors of DFS (**Table 5**).

**Table 5:** Univariable and multivariable analysis of DFS.

Variable	Univariable n = 154		Multivariable n = 150	
	HR (95% CI)	p value	HR (95% CI)	p value
Stage		** **		
I *vs* II-III-IV	13.4 (3.87–46.41)	**<0.0001**	6.11 (1.29–29.03)	**0.023**
Histology				** **
Endometrioid *vs* Serous	7.55 (0.95–59.65)	0.055	13.89 (1.33–145.5)	**0.028**
Myometrial invasion				
No infiltration or <50% *vs* >50%	2.27 (0.64–8.05)	0.204	1.4 (0.34–5.87)	0.643
Post operative tumor diameter (n=150)				
≤3.5cm *vs* >3.5cm	6.62 (1.71–25.63)	**0.006**	3.67 (0.75–17.99)	0.109
LVSI (n=151)				** **
Negative *vs* Positive	2.01 (1.03–12.35)	**0.044**	1.36 (0.31–5.9)	0.683
Uterine manipulator				** **
No *vs* Yes	0.64 (0.18–2.26)	0.484	0.92 (0.24–3.59)	0.903

Bold font highlights statistically significant differences. DFS, Disease Free Survival; HR, Hazard Ratio; CI, Confidence Interval; LVSI, Lymph Vascular Space Invasion.

At multivariable analysis, both stage and the histology had a significant influence on the DFS [HR (95%C I): 6.11 (1.29–29.03) and 13.89 (1.33–145.5), respectively]. The use or not of the uterine manipulator had no impact on DFS both at univariable and multivariable analyses [HR (95% Cl): 0.64 (0.18–2.26) and 0.92 (0.24–3.59), respectively].

## Discussion

This multicentric prospective randomized clinical trial demonstrates that the use of the intrauterine manipulator during minimally invasive hysterectomy for EC does not affect the rate and type of LVSI. Secondarily, our results suggest that the use of IUM does not increase the positive peritoneal cytology rate, and it does not influence the pattern of recurrence or the DFS in the study population. Of note, we found that the IUM may be associated with longer operation time and a marginally higher blood loss compared to operations accomplished without it.

The use of the intrauterine manipulator (IUM) has been considered for years as an important assistance in exposing the superficial and retroperitoneal anatomy during laparoscopic and robotic hysterectomies (9). In theory, this device is associated with shortening of the learning curve of standard laparoscopic hysterectomy (31), and it should be an aid to move minimal invasiveness beyond the standard laparoscopy in the ultra-minimally invasive field (32–35). Contrary to what was expected, our results showed a shorter operative time in the procedures carried out without IUM, particularly for laparoscopic procedure. Probably, this data may be explained by the high experience of involved surgeon and by the absence of the “manipulator’s installation time” (30). Interestingly, no differences in OT were recorded in the robotic population. The presence of the robotic platform (DaVinci Si/Xi) usually adds the “robot-specific time” to the OT, but the assistance of the fourth robotic arm may reduce the need of the uterine manipulation (8).

Despite this clear, practical advantage, the safety of the IUM in hysterectomy is still controversial and not confirmed by clinical evidence. Indeed, a recent review of the manufacturer’s characteristics of different types of uterine manipulators analyzed their safety and efficacy based on the technical features (36). The Authors underlined the paucity of data to demonstrate both the reduction of procedure-specific complications and the rate of IUM-related ones.

Our results confirmed that the use of IUM is not related to an increased risk of intraoperative complications (37). The only registered difference between the two arms regarded the EBL and the operative time of the laparoscopic procedure. However, the vascular complication that has been registered in arm B occurred during the pelvic lymphadenectomy, and it was obviously not related to the use of IUM.

When dealing with an intrauterine oncological disease, the use of IUM to handle the uterus has been considered to be involved in multiple phenomena with a potential influence on the oncological outcome and/or on postoperative treatment like (1) the transtubal tumor spread and (2) an increased rate of LVSI. In particular, Lim et al. and Sonoda et al. (17, 38) explained their findings of a higher incidence of positive peritoneal cytology when using the IUM with a retrograde seeding of tumor cells into the peritoneal cavity due to the pressure effect of the manipulator’s tip. Furthermore, Krizova et al. (39), in a blinded histopathologic review of 160 specimens of hysterectomies performed for malignant disease, described a significantly higher rate of lymph vascular pseudo-invasion and positive peritoneal cytology in those cases in which an IUM had been used.

Although these observations are logical and far from negligible, their real clinical meaning has been well clarified by several studies that suggested that the use of IUM is safe in EC staging (21, 40, 41). However, the retrospective nature of these researches did not allow the scientific community to say a definitive word on this issue.

In our results, no differences have been found in terms of LVSI status and peritoneal cytology between use and no use of IUM. Even the rate of focal and diffused lymph vascular embolism was comparable in both groups. This finding had been already shown by Lee et al. (29) in 2013 in a prospective randomized trial on 110 patients undergoing hysterectomy with and without the IUM. Authors concluded that despite the use of a uterine manipulator may favor the spread of tumor cells, it affects neither the LVSI nor the peritoneal cytology. However, the Authors acknowledged the short follow-up period (14 months), the heterogeneity of histotypes (endometrioid, serous, mucinous), and the differences in surgical approach (laparoscopy and robotics) as possible limitations of the study. Replying the data of previous literature (42), we did not detect any difference either in the rate of positive node or in the lymph nodal assessment technique in our study population. In particular, in our experience, the usage of the IUM did not influence the feasibility and success of the sentinel node mapping. However, we can hypothesize that in those cases in which a second indocyanine-green injection is needed (43), the presence of IUM may represent a concrete limitation.

More recently, Uccella et al. (23) drew the same conclusions with a multi-institutional retrospective analysis on a large population of 951 cases from seven different Italian hospitals. At the propensity-matched analysis, the Authors demonstrated the absence of an association between the use of a manipulator and the risk of recurrence. Those data have been confirmed in our analysis (**Table 5**). Again, in a recent retrospective study, Padilla-Iserte et al. reported, in a large uterine-confined EC series, that the use of IUM was associated with a worse oncological outcome in patients with uterus-confined endometrial cancer (Figo Stages I–II) who underwent minimally invasive surgery (44).

The heterogeneity of the study population in terms of histotype, stages, and the several models of IUM used in these two cited manuscripts could be considered a limitation in identifying in which subsets of patients the IUM can be safely used. Of note, Uccella et al. found an overall recurrence rate of 13.0%, which is much higher than our results. This discrepancy is mainly justified by the inclusion in these trials of type 2 EC, which has a much higher rate of relapses.

In our trial, in order to reduce any potential bias in interpreting the IUM role principally on the LVSI rate and secondly on surgical and oncological outcomes, only preoperative early-stage, endometrioid grade 1 or 2 EC patients were included, and one model of uterine manipulator was used. With a median follow-up time of 38.7 months, no statistically significant differences were found in terms of DFS, OS, recurrence rate, and pattern of recurrence. Moreover, the slight differences between the two arms merit to be widely argued: while in arm A three out of six (50%) recurrences were localized to the vaginal vault (**Table 4**), in the arm B the four recurrences were evenly distributed among the different categories. Even if this study was not powered to adequately investigate this aspect, despite the number of recurrences being very low, it could be reasonable to assume that the differences in exposing anatomy, and therefore the different handling of the uterus, could indirectly influence both the local radicality and the distant spread.

To the best of our knowledge, this is the largest randomized clinical trial focused on the influence of IUM in LVSI, in a specific and homogeneous subset of EC patients. Despite this, our study presents some limitations: the study designed was not powered to detect conclusive differences in oncology outcomes both regarding the rate of recurrences/deaths and the timing of events, so we could not draw definitive conclusions on the oncological impact of the use of IUM. For this reason, further powered and prospective experiences are needed to reinforce our results and confirm our assumptions.

## Conclusions

Assuming that the decision to use a uterine manipulator in EC staging is more a common-sense problem, its use should be considered as an important element of a personalized surgical treatment. Our results confirm that the intrauterine manipulator does not influence the LVSI status. Furthermore, our results suggest that IUM may not change the peritoneal cytology and not afflict the perioperative and oncological outcomes of early-stage EC patients undergoing laparoscopic/robotic staging. The accurate assessment of the preoperative risk-factors, like tumor dimensions, myometrial infiltration, and histotype (45, 46), should be done in parallel with the correct evaluation of the patient to avoid any surgical and oncological artifact.

## Data Availability Statement

The raw data supporting the conclusions of this article will be made available by the authors, without undue reservation.

## Ethics Statement

The studies involving human participants were reviewed and approved by Gemelli Hospital ethics committee (protocol number: 05152016). The patients/participants provided their written informed consent to participate in this study.

## Author Contributions

SA, SC, and GS designed the research. EP, CF, GM, SR, LT, FF, CR, VG, GV, AF, VC, AE, SU, AR, VAC, and FC performed the research. TP contributed analytic tools. SA, EP, GS, and CF wrote the paper. All authors contributed to the article and approved the submitted version.

## Conflict of Interest

The authors declare that the research was conducted in the absence of any commercial or financial relationships that could be construed as a potential conflict of interest.

## Publisher’s Note

All claims expressed in this article are solely those of the authors and do not necessarily represent those of their affiliated organizations, or those of the publisher, the editors and the reviewers. Any product that may be evaluated in this article, or claim that may be made by its manufacturer, is not guaranteed or endorsed by the publisher.
